# Saving time and money in biomedical publishing: the case for free-format submissions with minimal requirements

**DOI:** 10.1186/s12916-023-02882-y

**Published:** 2023-05-10

**Authors:** Amy Clotworthy, Megan Davies, Timothy J. Cadman, Jessica Bengtsson, Thea O. Andersen, Manik Kadawathagedara, Johan L. Vinther, Tri-Long Nguyen, Tibor V. Varga

**Affiliations:** 1 Section of Epidemiology, Department of Public Health, University of Copenhagen, Bartholinsgade 6Q, DK-1356 Copenhagen K, Denmark; 2 Center for Healthy Aging, Faculty of Health and Medical Sciences, University of Copenhagen, Copenhagen, Denmark

**Keywords:** Publishing, Journal article, Formatting, Submission guidelines, Policy

## Abstract

**Background:**

Manuscript preparation and the (re)submission of articles can create a significant workload in academic jobs. In this exploratory analysis, we estimate the time and costs needed to meet the diverse formatting requirements for manuscript submissions in biomedical publishing.

**Methods:**

We reviewed 302 leading biomedical journals’ submission guidelines and extracted information on the components that tend to vary the most among submission guidelines (the length of the title, the running title, the abstract, and the manuscript; the structure of the abstract and the manuscript, number of items and references allowed, whether the journal has a template). We estimated annual research funding lost due to manuscript formatting by calculating hourly academic salaries, the time lost to reformatting articles, and quantifying the total number of resubmissions per year. We interviewed several researchers and senior journal editors and editors-in-chief to contextualize our findings and develop guidelines that could help both biomedical journals and researchers work more efficiently.

**Results:**

Among the analyzed journals, we found a huge diversity in submission requirements. By calculating average researcher salaries in the European Union and the USA, and the time spent on reformatting articles, we estimated that ~ 230 million USD were lost in 2021 alone due to reformatting articles. Should the current practice remain unchanged within this decade, we estimate ~ 2.5 billion USD could be lost between 2022 and 2030—solely due to reformatting articles after a first editorial desk rejection. In our interviews, we found alignment between researchers and editors; researchers would like the submission process to be as straightforward and simple as possible, and editors want to easily identify strong, suitable articles and not waste researchers’ time.

**Conclusions:**

Based on the findings from our quantitative analysis and contextualized by the qualitative interviews, we conclude that free-format submission guidelines would benefit both researchers and editors. However, a minimum set of requirements is necessary to avoid manuscript submissions that lack structure. We developed our guidelines to improve the *status quo*, and we urge the publishers and the editorial-advisory boards of biomedical journals to adopt them. This may also require support from publishers and major international organizations that govern the work of editors.

**Supplementary Information:**

The online version contains supplementary material available at 10.1186/s12916-023-02882-y.

## Background

Since the introduction of the first printed journal in 1665, the intent behind publishing papers has been to advance scientific knowledge by building on other scholars’ results and avoiding duplications [1]. The structured format that developed over time has enabled the systematic recording and archiving of scientific knowledge [2–5]. However, with the advent of digital and online formats, the need to format manuscripts according to a journal’s specific layout for a printed issue (which was often dictated by the press being used) has become obsolete. Although the digital format has improved access, searchability, and navigation within and between journal articles, it has not substantially changed the form of the scholarly journal [6].

When academic authors currently format a manuscript to adhere to a journal’s submission guidelines, it means that their documents should arrive in editorial in-boxes with a specific structure and length, following certain formatting for figures and tables, author information, reference style—the list goes on. In theory, the main benefit of such standardized formatting is that it facilitates the evaluation process at both the editorial and the peer-review stage [7]. Several prominent journals even warn authors that failure to comply with their specified guidelines may result in a rejection of a submitted article (or its return to the authors), which may significantly delay the publishing process [8–11].

However, initial manuscript submission guidelines vary substantially among journals. This places a time constraint and financial strain on authors in terms of formatting and reformatting their articles [12]. Informally, authors have raised numerous issues concerning the current complexity of journal systems, processes, and guidelines. Stressing the amount of time spent on the process of formatting and submitting manuscripts, authors have expressed a preference for (1) clean, simple manuscript-submission guidelines, (2) standardized formatting guidelines, and (3) universal online-submission systems [13].

As the problematic nature of varying submission requirements has been raised several times [14–19] and reported to be a significant burden for authors [13], some journals have developed their own standardized templates, moved towards “free-format” first submissions, or may be willing to evaluate papers posted on preprint servers [20] to simplify the process. Despite these improvements, the overall academic-publishing industry has experienced only a marginal shift, with publishers/journals taking individual action rather than collaborating on a shared solution [1]. We believe that these sporadic improvements are laudable; however, varying submission requirements will continue to put time and financial burden on researchers and slow the process of scientific dissemination until a single unifying solution to streamline manuscript submission is found.

In our exploratory analysis, we reviewed 302 leading biomedical journals’ submission requirements to estimate the time and costs needed to meet the diverse formatting requirements for an initial submission in biomedical publishing. To gain insight into the processes involved, we sought the experiences and perspectives of not only authors but also journal editors. Based on our findings from the quantitative study and the subsequent qualitative contextualization, in this article, we propose concrete suggestions for journal guidelines that will both decrease the time needed for submission and reduce the financial burden the current system imposes on academia.

## Methods

To review current formatting requirements, we extracted a list of the top 1000 academic journals [21] based on their 2019 CiteScore values, a metric used to measure the average citations per document in a particular journal over a certain period of time [22]. We excluded journals that do not publish original research articles (*n* = 181) and, from the remaining list, all that are non-biomedical journals (*n* = 427); we present the detailed selection process in Additional file 1: Text S1. We then sorted the remaining list (*n* = 392) by decreasing CiteScore values and included the top 302 biomedical journals for review. The 302 journal titles were divided among all co-authors, who then manually extracted the eight components that we found to vary the most among submission guidelines: the maximum length of the title, running title, abstract, and manuscript text; the structure of the abstract and the manuscript; the maximum number of items (tables and/or figures) and references allowed; and the availability of a submission template on the journal’s website (Additional file 2: Table S1). If questions arose, the specific co-author and the senior author discussed and resolved them. The co-authors calculated the number of possible formats by computing the number of possible combinations of the eight criteria on which we focus in our review.

We extracted average annual salaries in the European Union and the United States for PhD students, postdocs, assistant professors, associate professors, and full professors, and we averaged these values to calculate an *average researcher salary* in these regions [23–25]. Using an estimate of ~ 1750 work hours per year, which is the Organisation for Economic Co-operation and Development (OECD) average for the European Union and the United States [26], we calculated average hourly salaries. Using the four hours of reformatting time per article that Sobani et al. estimated in a previous report [15] and, assuming that each article is desk-rejected and resubmitted once (on average), also proposed by Sobani et al. [15], we estimated the cost of reformatting one article. We then extracted the number of entries published in PubMed each year between 2000 and 2021 [27]. Based on the salary estimates and the number of articles, we estimated the annual financial loss between 2000 and 2021. We then used a time-series prediction using quadratic models with years as a predictor using the *lm*() function in R (v.4.2.2) [28] to extrapolate financial loss between 2022 and 2030.

We also conducted semi-structured qualitative interviews with four mid-career researchers and four senior journal editors or editors-in-chief between 19 July and 18 August 2022. Seven interviews were conducted via secure video calls, while one researcher provided email comments to our list of questions. All of the interviews were conducted in English; for seven out of eight of the participants, English was their primary working language but not their first language (i.e., native tongue). The pre-determined interview questions and follow-up questions were developed by (i) surveying co-authors for suggestions and (ii) informally soliciting suggestions from other colleagues during departmental seminars. The most relevant quotes from the interviews were transcribed, and all personally identifying features were removed from the transcripts. The complete process of the qualitative interviews is described in Additional file 3: Text S2 [29–35].

## Results

### Diversity in formatting requirements

In our analysis, we identified significant diversity in initial formatting requirements. For each category, a substantial proportion of journals did not specify requirements (or we were unable to locate them). In particular, the following percentage of journals did not indicate information related to running titles and their length (65%), length of the title (64%), maximum number of references (58%), number of figures and tables (48%), main text length (34%), manuscript structure (27%), abstract structure (21%), and abstract length (14%). The lack of information regarding these parameters did not necessarily promote free-format submission; for example, we differentiated between journals explicitly stating no word limit for the main text (3%) and those for which this information was not available (or we were unable to locate it) (34%). This high percentage of missing information suggests that submission guidelines are incomplete and/or difficult to navigate, adding an additional burden to the already cumbersome process of (re)formatting a manuscript.

**Fig. 1:**
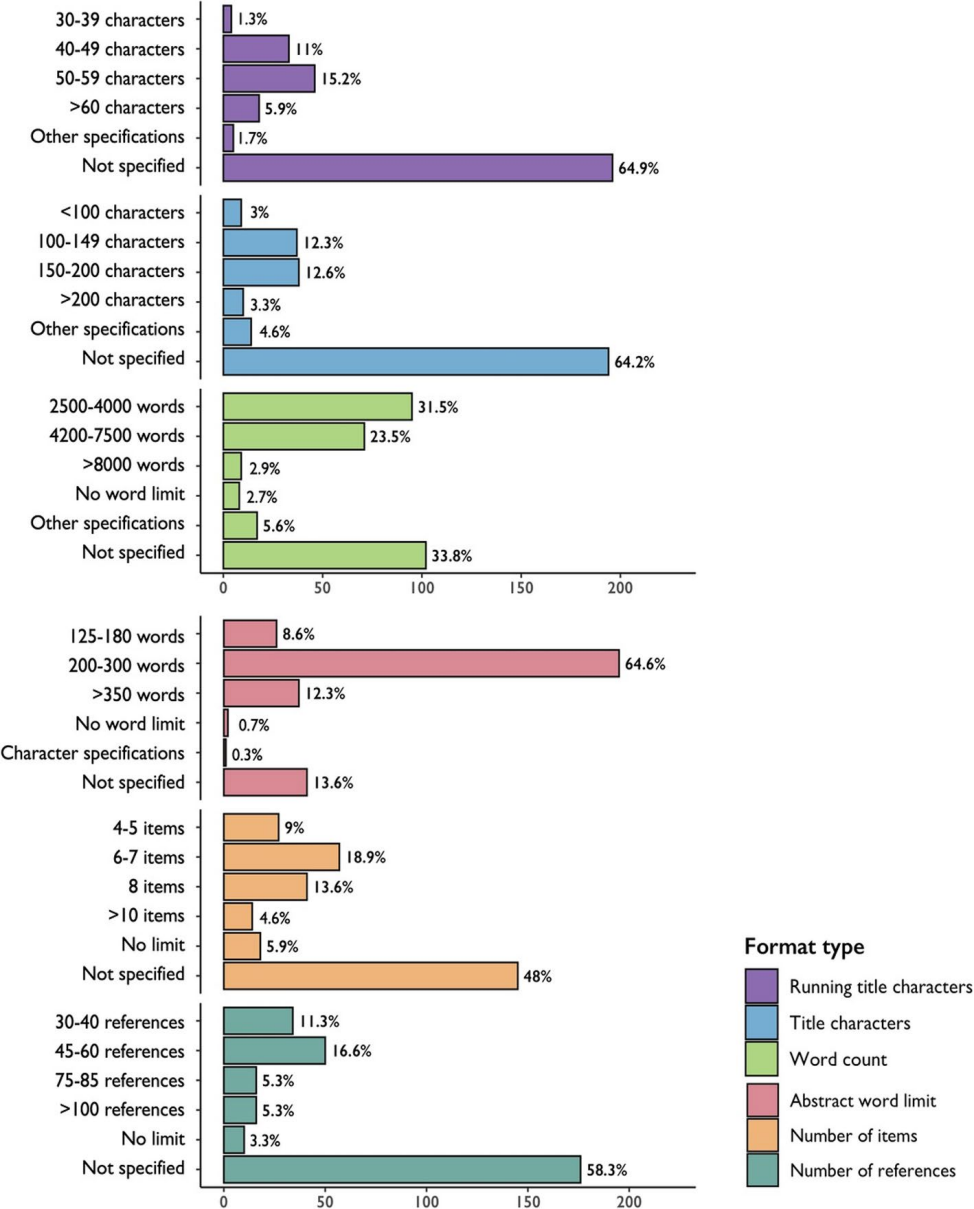
Diversity in submission requirements based on the review of leading biomedical journals (*N* = 302)

**Fig. 2:**
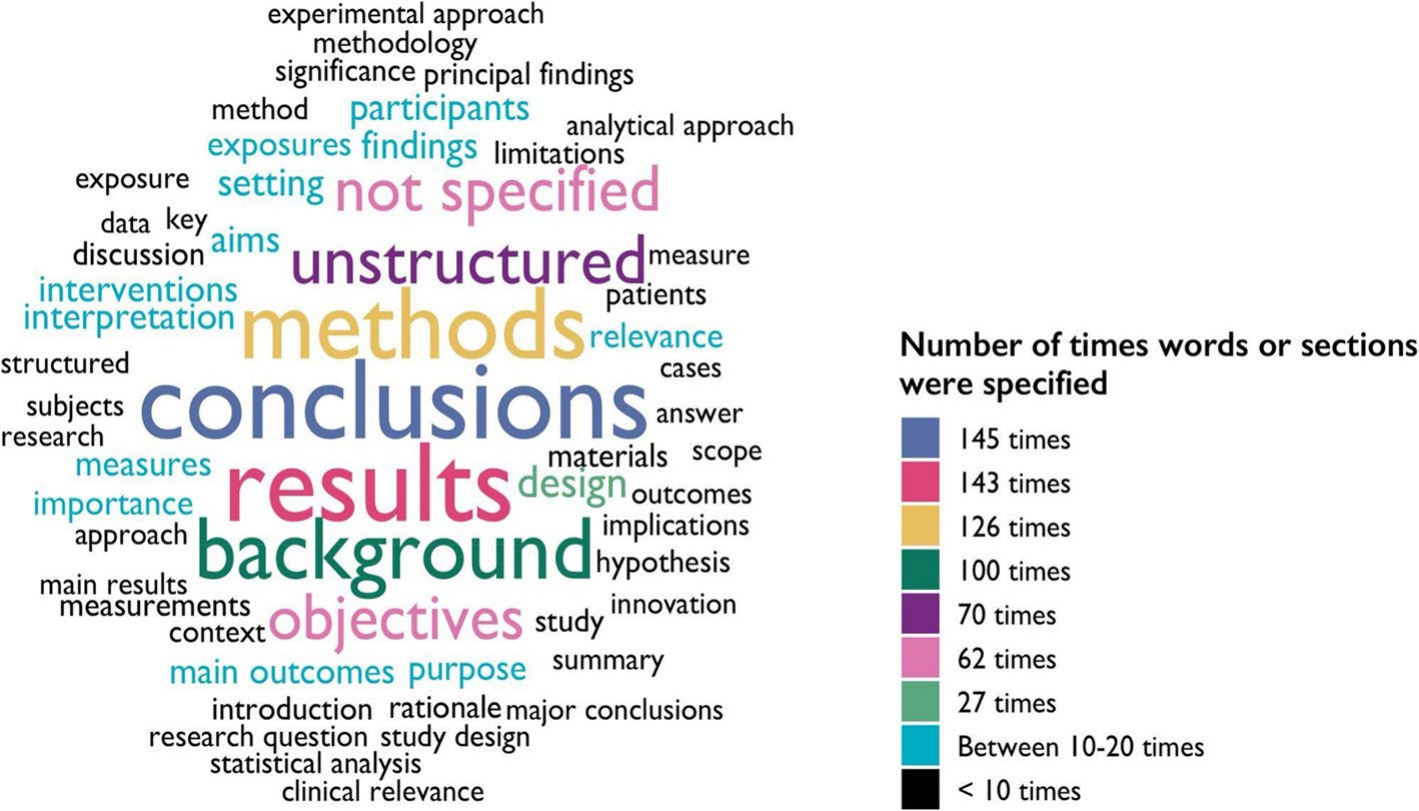
Word cloud of abstract sections in leading biomedical journals (*N* = 302)

**Fig. 3:**
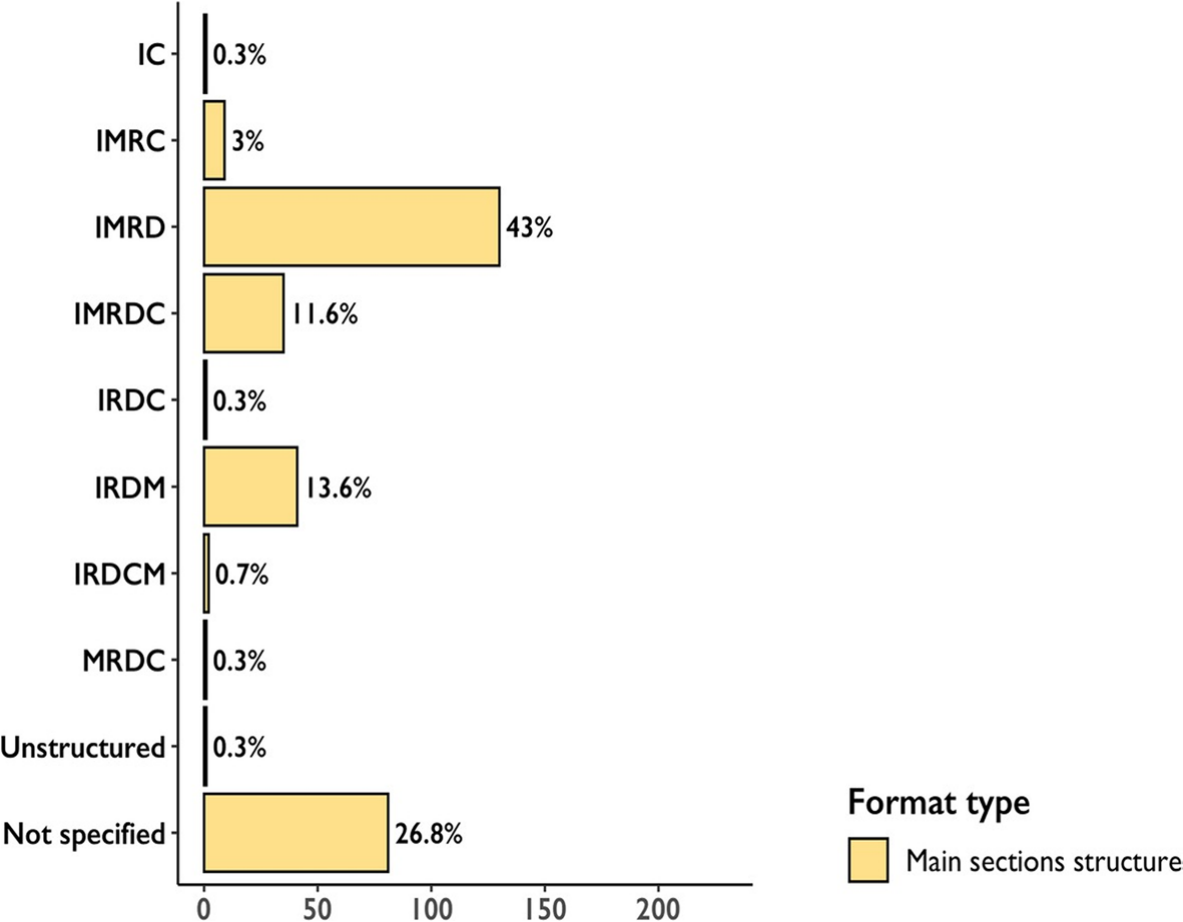
Main text structures in leading biomedical journals (*N* = 302). C—Conclusions; D—Discussion; I—Introduction; M—(Materials and) Methods; R—Results

We categorized the requirements for maximum length and overall structure; where these were specified, running-title lengths tended to be between 50 and 59 characters (15%), title lengths were between 150 and 200 characters (13%), abstract word limits were between 200 and 300 words (65%), main text word limits were between 2500 and 4000 words (32%), the number of items were 6–7 (19%), and the number of references were between 45 and 60 (17%) (Fig. 1). With regard to abstracts, 21% did not specify a structure (or we did not find it), 22% required unstructured abstracts, and the remaining 57% required a structured abstract, with Background, Methods, Results, and Conclusions being the most frequently specified sections (Fig. 2). In terms of manuscript structure, the Introduction, Methods, Results, and Discussion format (known as IMRaD) was the most frequently specified (43%) (Fig. 3). While journals belonging to the same family (e.g., *Journal of the American Medical Association* [*JAMA*] family, *Science* family) usually follow the same formatting conventions, the vast majority of journals we analyzed had a unique set of requirements. We acknowledge that the selection process of leading biomedical journals (i.e., those with the highest CiteScore values) might bias our findings; however, we find it unlikely that selecting journals with lower average scores or selecting journals according to other criteria would significantly impact our conclusions regarding either the diversity or the variation in submission requirements.

In our calculation of the number of possible formats, we obtained a staggering number of ~ 6.3 billion possible unique sets of formatting requirements. In the next section, we review and estimate the financial cost of reformatting articles for a new submission.

### The cost of (re)formatting articles

**Fig. 4:**
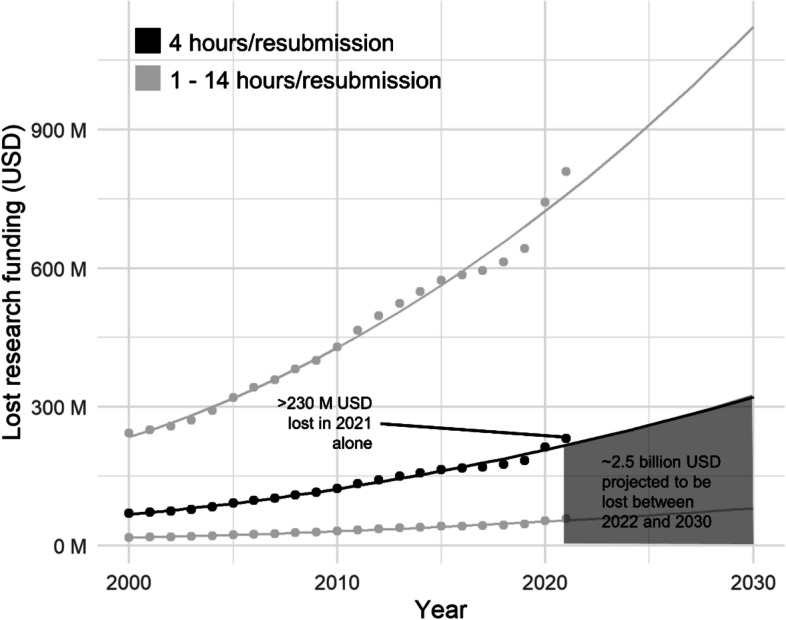
The cost of reformatting manuscripts after editorial rejections. We estimated the cost of reformatting one original research article to be approximately 130 USD. The number of entries published in PubMed was extracted for each year between 2000 and 2021. We assumed that each manuscript needs to be reformatted once (on average) before submission to another journal. Between years 2022 and 2030, we predicted the lost research funds using a quadratic model with calendar year as predictor. The error bound was calculated using a previously reported minimum estimate of one hour and a maximum estimate of 14 h per article

We obtained average annual salaries in the European Union and the United States for PhD students (33,000 USD), postdoctoral researchers (43,000 USD), assistant professors (56,000 USD), associate professors (67,000 USD), and full professors (86,000 USD), and then calculated an *average researcher salary* of 57,000 USD. With an estimate of ~ 1750 work hours per year, we arrived at an average hourly salary of 32.6 USD. Using four hours of reformatting time, we estimated the cost of reformatting one article to be approximately 130 USD. In 2000, the number of published entries on PubMed was ~ 0.5 million, while in 2021, a record number of ~ 1.8 million were published. Assuming one rejection per paper (on average), we calculated that ~ 230 million USD were lost in 2021 alone. Should the current practice remain unchanged within this decade, we estimate ~ 2.5 billion USD could be lost between 2022 and 2030—solely due to reformatting articles after a first editorial desk rejection. These estimates are depicted in Fig. 4, which also shows projected losses with an error bound based on a previously reported minimum estimate of one hour [14, 16] and a maximum estimate of 14 h per article [12]. The extrapolated financial loss for the period 2022–2030 roughly equates to 75 million hours of lost time. Calculating based on 1750 work hours per year, this means that there could be a cumulative delay of approximately 43,000 years in the public dissemination of results—solely due to the reformatting of papers from 2022 to 2030.

## Discussion

### Our results in context

For many authors, the first step in publishing a scientific article is identifying the most appropriate journal(s) to disseminate their research, and then formatting the manuscript according to the target journal’s submission guidelines. The percentage of papers rejected without peer-review can vary across journals, disciplines, and publishing models, with some journals rejecting more than two-thirds of all first submissions at the editorial level [36]; this is known as a ‘desk reject’. When an article is returned to the corresponding author, they often want to resubmit to another journal quickly to avoid delays in dissemination. Once the author identifies another appropriate journal, they must then understand its specific submission requirements. It is at this stage that reformatting can be particularly cumbersome and time-consuming; numerous tasks are involved in reformatting manuscripts to adhere to the second journal’s specific requirements, as well as additional tasks related to the submission process itself.

Sobani et al. estimated that the total time used to resubmit one article is ~ four hours for submitting authors [15]. Others have estimated that reformatting a single manuscript can take from one to 14 h or more [12, 14, 16]. In practice, the time needed to reformat can be substantially longer, especially when the resubmission requires substantive reductions to the manuscript’s word count. For instance, an article rejected by *Science* (max. 4500 words) cannot be immediately resubmitted to *The Lancet* (max. 3500 words) or *The New England Journal of Medicine* (max. 2700 words). Such substantive differences in length also exist among the specialized journals, a specific barrier that was mentioned in our interviews. One researcher said, “*For a typical manuscript, [reformatting takes] a couple of days … If you have to rewrite a lot because you have to cut 2,000 words, then it probably takes more time*”.

When significant rewriting takes place, the corresponding author will typically circulate a revised manuscript to any co-authors in order to obtain their approval for the new submission. In these cases, one of the researchers said, “*It could take a week [because] if we need to add an extra section, then we need to talk again between the different authors*”. Reducing the word count is not the only challenge. Often, additional changes need to be implemented throughout the manuscript; e.g., the number of figures and tables, adjustments to the abstract text/structure, reference style, or the title. As one researcher put it, “*When you need to reformat, it takes forever, because sometimes [journals] are really picky with some detail that you really have to follow, and they won’t send it out to review if it doesn’t fulfil their formatting criteria*”. However, we did find that approximately 11% of the journals in our study provided a template on their website to guide authors with formatting.

Our estimate of the financial loss per article was lower than others found in the literature. A report by LeBlanc et al. surveyed ~ 400 individuals on the topic of resubmitting papers and arrived at an estimate of 477 USD lost per paper. This estimate took into account 14 h of work per manuscript and a median number of two resubmissions per paper based on survey responses [12]. Using this estimate, they calculated a loss of 1908 USD per year in research funding for each author. Sobani et al. estimated 2.6 million hours of lost academic work per year based on half a million biomedical articles annually, three hours of resubmission time, and one rejection per submitted article [15]. Another report by Khan et al. arrived at approximately 1.5 million hours lost in reformatting rejected articles per year, based on an average rejection rate of 62% for 2.5 million published papers every year, and one hour spent on reformatting time per manuscript [14]. Single author Budd estimated the time lost due to resubmission after editorial rejection by the journal *Nature* alone (desk-rejection rate: 92%). They concluded ~ 10,000 h of work time were lost per year, based on one hour of work per article to be resubmitted elsewhere [16]. Finally, Jiang et al. estimated the annual cost of reformatting manuscripts to be ~ 1.1 billion USD, a fivefold increase on our calculations [37].

We believe that our calculations are conservative estimates; the real cost in lost time and money is likely to be higher. Research is a collaborative process, and reformatting articles results in wasted time for everyone in the finite academic ecosystem, including all authors, peer-reviewers, editors, publishers, and ultimately, the public.

Thus far, we have shown that reformatting manuscripts takes an enormous toll on the research ecosystem in terms of both time and money. Next, we propose a simple solution that could help to prevent these projections from being realized.

### Potential routes to decrease the time and financial burden of submissions

**Fig. 5:**
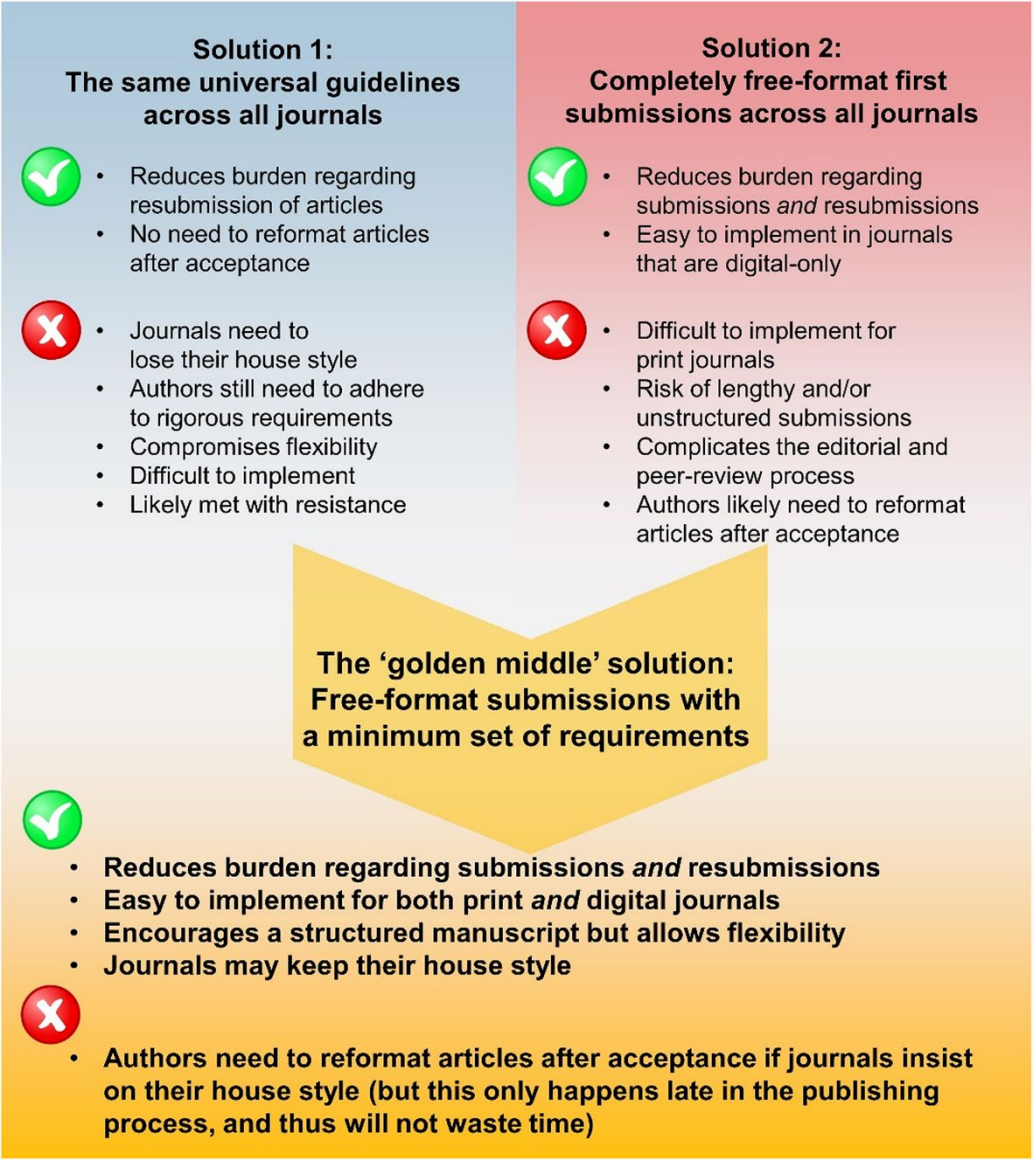
The two possible avenues for simplifying the submission process and our proposed solution: free-format initial submissions with a minimum set of requirements

Based on our investigation, we have identified two possible solutions (Fig. 5) that could significantly decrease the time and money lost due to heterogeneous initial-submission guidelines. The first suggestion is to adopt the *same* universal guidelines across all biomedical journals. The second is to remove the requirement for specific formatting at first submission; in this case, journals could request specific formatting guidelines but only *after* acceptance [14].

Both solutions have benefits and drawbacks. While universal guidelines would drastically reduce the burden on authors, this approach could force journals to lose their distinctive ‘look’ (house style) as well as remove flexibility specific to their subject matter or print format. But, in practice, it is unlikely to impact the editorial process. For example, one editor told us: “*There are journals that in principle have formats but don’t care. […] The editor hardly ever cares about how long [the text] is or how many figures it has on first instance*”. The introduction of universal submission guidelines could also be a costly endeavor for journals/publishers, which could lead to resistance, as another editor noted: “*It would be a very high lift to get all publishers together and agree on universal submission guidelines. And people use different submission systems that have different dependencies. […] If you’re an editor at one journal, you’ll know that in a way we’re at the tyranny of very clunky submission systems that are not very good. […] Changing that is very costly for individual publishers.*”

The second option of a free-format first submission is now easier than ever. An increasing number of journals are published online-only, so articles in these journals require less post-processing upon acceptance, and there are fewer restrictions on length. Indeed, the editors we interviewed indicated that there is an increasing trend towards free-format initial submission. One said, “*My understanding [is that] less and less journals […] have rigid format restrictions. I think most journals are […] moving towards initial-submission agnostic views*”. Another said, “*Some journals have introduced what they call ‘hassle-free’ submission [and] our journal is moving towards it*”. Despite this development, the number of journals with free-format initial submissions is still estimated at only around ~ 5% of all biomedical journals [37].

With regard to a free-format approach, several editors raised concerns about the burden of having to review papers that were unreasonably long, poorly structured, or just badly written. For example, one editor noted, “*Sometimes you get submissions […] which are, basically, somebody’s whole PhD thesis just dumped there*”. Another editor said: “*Every so often somebody sends me an article that […] looks like […] somebody had a dictaphone in their hand and just spoke to it for 20 minutes and got someone to transcribe it*”. For a format-free approach to be successful, this same editor reflected that, “*It would depend on everybody submitting having a vague sense of what a scientific article looks like*”. Thus, the key point in favor of a free-format approach was nicely summarized as such: “*I like the articles to be standardly structured. But there is flexibility around that. It doesn’t always have to be the same. It should be recognizable as a scientific article*.”

Considering both options and taking the experiences of both researchers and journal editors into account, we propose a ‘golden middle’. Specifically, we believe that free-format submission guidelines are the way forward, but a minimum set of structural requirements is necessary to avoid the submission of manuscripts that are excessively long or simply not “*recognizable as a scientific article*”.

Our proposed minimum set of requirements, presented in Table 1, was developed based on the current submission requirements of the 302 biomedical journals we reviewed as well as on our interviews with the editors. We propose no requirements regarding titles, running titles, title pages, abstract structure, item formatting, maximum number of items, maximum number of references, or reference styles at first submission—we believe that these details can be addressed later during the ‘revise and resubmit’ stage, or even after acceptance. As we learned from our interviews with the editors, it is at the resubmission stage when most journals tend to commit to shepherding manuscripts towards acceptance and publication: “*Where we do a lot of checking is when the revised version comes back in. Because we have a strong commitment to revisions*”. Similarly, another editor said, “*We don’t bother with any format until we are inviting for revision. When we invite for revision, we are pretty confident. […] After peer-review, that’s when we make a commitment.*”

**Table 1:** A minimum set of requirements for the first submission of an original research article

• Abstract of maximum ~ 300 words that includes information on the project background, methods, results, and conclusions
• Main body of maximum ~ 3000–5000 words, with sections that include information on the project background, methods, results, and discussion and contextualization of findings
• If the manuscript differs substantially from these requirements, please explain the reasons for this to the managing editor (e.g., via the cover letter or the comment box during submission)

As outlined in Table 1, even the two requirements that we propose allow for flexibility. In cases where a journal may have additional specific requirements/sections (e.g., clinical relevance, key messages, graphical abstract), we encourage them to ask authors to upload these parts in separate files to keep the main manuscript easy to resubmit to another journal. If print journals with space constraints need to enforce word limits that are stricter than those defined in our minimum set of requirements, then we propose that these journals still adopt the same basic requirements and also that they should also be willing to review longer papers. However, they should clearly explain to the submitting author(s) that word count will need to be reduced upon acceptance in order to adhere to their pre-specified maximum lengths.

Furthermore, we recommend that online content-management systems incorporate auto-fill tools that require an initial upload of the manuscript file and then use the available information to populate fields in the system. Manually copying titles, abstracts, and key words as well as filling in authorship and funding information can substantially prolong the submission process. For example, one researcher explained, “*In the submission system, there are a lot of steps that we need to go through. […] You have to write the name, the address—things that you already have on the first page of the article. […] And the names are not recognized by the system. These are unnecessary things that really waste time.*” These manual processes can also be prone to error, especially when manuscripts have many authors and/or funders. Oftentimes, following a submission of a manuscript, all co-authors need to register their profile on the journal-submission site, provide information related to expertise, keywords, and fill in other fields; e.g., the newly introduced mandatory diversity and inclusion surveys (which we fully support—after acceptance), and then proceed to approve the corresponding author’s submission. When a manuscript has multiple co-authors, such processes can cause significant delays. One of the editors we interviewed had had similar experiences on both sides of the editorial desk, explaining, “*I’ve published articles […] where it is a bloody pain trying to work it out how to format it for [a journal], and jump through the ludicrous hoops of their content-management system. […] I’ve used most of the content-management systems or editorial management systems, and found that all of them tripped me up at various points*”. Artificial-intelligence tools already implemented by some journals can automatically recognize and process these sections, which can save significant time for the corresponding author. If journals do not have the means to incorporate such tools, then we suggest requesting this detailed information only at the resubmission/acceptance stage.

From our interviews, we were struck by how much alignment there was between the editors and the researchers. As we learned, researchers/authors would like the submission process to be as straightforward and simple as possible, and editors want to easily identify strong, suitable articles and not waste the authors’ time. Most editors emphasized that the manuscript’s scientific content and its relevance to the journal’s aims and scope were far more important than any formatting details. As one editor put it: “*I didn’t care what format they were in, I just cared about the science in the paper.*”

## Conclusions

Based on this study, we urge publishers and the editorial-advisory boards of biomedical journals—who have the mandate to make these changes—to follow the increasing trend to adopt free-format initial submissions and to implement our proposed minimum set of requirements. In addition, we recommend that influential associations insist that publishers and journals adopt free-format first submissions with our proposed minimum set of requirements. Organizations that are likely to have a vested interest in the changes we are proposing are editorial associations (e.g., International Committee of Medical Journal Editors [ICMJE], World Association of Medical Editors), academic and university organizations (e.g., Association of American Universities, League of European Research Universities), and certain funding bodies (e.g., National Institutes of Health [NIH], the European Research Council [ERC], and the Wellcome Trust). Most important, large publishing houses are in the strongest position to make a meaningful impact by implementing our proposed guidelines across their portfolios of hundreds of journals.

We are not the only ones demanding this change. As one editor noted, “*There’s a clamour [for reform] on Twitter. Every week there’s stuff going on about formats and submission. People do want them to be liberalized [because] it’s a waste of time*”. Based on the results of our study, we believe now is the time to act and spark institutional change in the scientific community that will ultimately enable researchers and editors alike to use their precious time to advance science and not endlessly format papers. We would like to stress that we do not call for the abolition of journals’ unique house styles, formats, and designs; we simply believe that the simplified process of submissions that we propose here would drastically reduce the time and financial demands being experienced by an already overburdened research ecosystem. As one editor put it: “*Just send [the article] in to us, and we’ll take a look at it. And if we like it, we’ll send it out for peer review. And if it meets peer review, we’ll send it back to you. And THEN you can put it into our journal’s format. I think that’s respectful of the authors’ time and effort.*”

## Supplementary Information

**Additional file 1: Text S1.** Selection process of the 302 biomedical journals for quantitative analysis. This document outlines the selection process used to identify the journals included in our quantitative analysis. It provides a list of all the journal categories that were excluded and describes how we arrived at our final selection of 302 journals that were included in the analysis.

**Additional file 2: Table S1.** Initial submission guidelines for leading biomedical journals. This anonymized table presents the quantitative statistics extracted from the 302 selected biomedical journals. The data we collected from these journals include the maximum character count for the title and running title, the maximum word count for the abstract and manuscript body, the maximum number of items, the maximum number of references, abstract sections, main sections, and whether a template is available for authors.

**Additional file 3: Text S2.** Interview protocol and questions for researchers and editors. This document contains a detailed description of the interview protocol used for the researchers and journal editors, including ethical considerations related to the qualitative aspect of our study and the informed consent form. The file also includes email templates that were sent to the researchers and editors we attempted to interview. It outlines the questions asked of the informants, as well as any follow-up questions.

## Data Availability

All data used in our analyses are available in the Supplemental Material.

## Abbreviations

ERC: European Research Council
ICMJE: International Committee of Medical Journal Editors
IMRaD: Introduction, Methods, Results, and Discussion format
JAMA: Journal of American Medical Association
NIH: National Institutes of Health
OECD: Organisation for Economic Co-operation and Development
