# Supplementary material for: Saving time and money in biomedical publishing: the case for free-format submissions with minimal requirements
Source: BMC Med. 2023 May 10;21:172. doi: 10.1186/s12916-023-02882-y (PMC10170849; doi:10.1186/s12916-023-02882-y)
Supplement: Supplementary file 1 — Additional file 1: Text S1. Selection process of the 302 biomedical journals for quantitative analysis. This document outlines the selection process used to identify the journals included in our quantitative analysis. It provides a list of all the journal categories that were excluded and describes how we arrived at our final selection of 302 journals that were included in the analysis. [file 12916_2023_2882_MOESM1_ESM.docx]

**Text S1. Selection process of the 302 biomedical journals for quantitative analysis.**

We obtained a dataset of 1000 journals from Kaggle (Impact factor of top 1000 journals; <https://www.kaggle.com/datasets/umairnasir14/impact-factor-of-top-1000-journals>

We excluded the following:

**Journals with the word “REVIEW” in title (n=156)**

**Journals with the word “CONFERENCE” in title (n=5)**

**Journals that exclusively publishes surveys (n=5)**

**Journals that exclusively publishes letters (n=15)**

**Non-biomedical journals (n=427)** categorized as follows:

- Accounting (n=2)
- Acoustics and Ultrasonics (n=1)
- Aerospace Engineering (n=2)
- Agricultural and Biological Sciences (n=1)
- Agronomy and Crop Science (n=3)
- Analytical Chemistry (n=5)
- Analysis (n=1)
- Animal Science and Zoology (n=2)
- Applied Microbiology and Biotechnology (n=2)
- Applied Mathematics (n=6)
- Aquatic Science (n=2)
- Artificial Intelligence (n=3)
- Arts and Humanities (n=2)
- Astronomy and Astrophysics (n=2)
- Atmospheric Science (n=4)
- Atomic and Molecular Physics, and Optics (n=8)
- Automotive Engineering (n=3)
- Biomaterials (n=2)
- Biomedical Engineering (n=3)
- Biophysics (n=6)
- Biotechnology (n=4)
- Building and Construction (n=5)
- Business and International Management (n=7)
- Catalysis (n=1)
- Civil and Structural Engineering (n=5)
- Communication (n=1)
- Computational Mathematics (n=3)
- Computational Mechanics (n=1)
- Computational Theory and Mathematics (n=1)
- Computer Graphics and Computer-Aided Design (n=3)
- Computer Networks and Communications (n=9)
- Computer Science (n=2)
- Computer Science Applications (n=10)
- Computer Vision and Pattern Recognition (n=1)
- Condensed Matter Physics (n=5)
- Control and Optimization (n=1)
- Control and Systems Engineering (n=9)
- Development (n=1)
- Earth-Surface Processes (n=1)
- Ecology (n=6)
- Economics and Econometrics (n=14)
- Economics, Econometrics and Finance (n=1)
- Education (n=7)
- Electrical and Electronic Engineering (n=9)
- Electrochemistry (n=3)
- Electronic, Optical and Magnetic Materials (n=2)
- Energy Engineering and Power Technology (n=10)
- Engineering (n=2)
- Environmental Chemistry (n=1)
- Environmental Engineering (n=4)
- Filtration and Separation (n=1)
- Finance (n=1)
- Fluid Flow and Transfer Processes (n=2)
- Forestry (n=1)
- Fuel Technology (n=1)
- General Business, Management and Accounting (n=1)
- General Chemical Engineering (n=8)
- General Chemistry (n=8)
- General Computer Science (n=4)
- General Earth and Planetary Sciences (n=3)
- General Economics, Econometrics and Finance (n=1)
- General Energy (n=1)
- General Engineering (n=8)
- General Environmental Science (n=5)
- General Materials Science (n=9)
- General Mathematics (n=4)
- General Pharmacology, Toxicology and Pharmaceutics (n=1)
- General Physics and Astronomy (n=5)
- General Social Sciences (n=2)
- Geochemistry and Petrology (n=1)
- Geography, Planning and Development (n=4)
- Geology (n=5)
- Hardware and Architecture (n=4)
- Health (social science) (n=4)
- Health Policy (n=4)
- Human Factors and Ergonomics (n=1)
- Human-Computer Interaction (n=2)
- Industrial and Manufacturing Engineering (n=6)
- Information Systems (n=12)
- Information Systems and Management (n=3)
- Instrumentation (n=3)
- Law (n=1)
- Library and Information Sciences (n=2)
- Life-span and Life-course Studies (n=1)
- Management Information Systems (n=1)
- Management of Technology and Innovation (n=1)
- Management Science and Operations Research (n=4)
- Management, Monitoring, Policy and Law (n=1)
- Marketing (n=2)
- Materials Chemistry (n=1)
- Materials Science (n=2)
- Mechanical Engineering (n=8)
- Mechanics of Materials (n=1)
- Media Technology (n=3)
- Metals and Alloys (n=3)
- Modeling and Simulation (n=7)
- Nature and Landscape Conservation (n=2)
- Nuclear and High Energy Physics (n=3)
- Nuclear Energy and Engineering (n=1)
- Organic Chemistry (n=4)
- Organizational Behavior and Human Resource Management (n=2)
- Physical and Theoretical Chemistry (n=2)
- Physics and Astronomy (n=1)
- Plant Science (n=12)
- Political Science and International Relations (n=1)
- Pollution (n=2)
- Polymers and Plastics (n=6)
- Renewable Energy, Sustainability and the Environment (n=3)
- Safety, Risk, Reliability and Quality (n=2)
- Signal Processing (n=2)
- Social Psychology (n=1)
- Social Sciences (n=4)
- Sociology and Political Science (n=5)
- Software (n=3)
- Soil Science (n=2)
- Space and Planetary Science (n=4)
- Spectroscopy (n=1)
- Statistical and Nonlinear Physics (n=3)
- Statistics and Probability (n=2)
- Strategy and Management (n=5)
- Structural Biology (n=2)
- Surfaces, Coatings and Films (n=3)
- Theoretical Computer Science (n=3)
- Transportation (n=1)
- Waste Management and Disposal (n=1)
- Water Science and Technology (n=2)

After exclusions, we had a list of n=392 journals.

We sorted this list by descending CiteScore values and reviewed the top 302 journals. In case journals were identified as non-eligible according to our previous exclusion criteria (e.g., not publishing original research articles or non-biomedical) during the analysis phase, these journals were discarded from the analysis.
