# Supplementary material for: Saving time and money in biomedical publishing: the case for free-format submissions with minimal requirements
Source: BMC Med. 2023 May 10;21:172. doi: 10.1186/s12916-023-02882-y (PMC10170849; doi:10.1186/s12916-023-02882-y)
Supplement: Supplementary file 3 — Additional file 3: Text S2. Interview protocol and questions for researchers and editors. This document contains a detailed description of the interview protocol used for the researchers and journal editors, including ethical considerations related to the qualitative aspect of our study and the informed consent form. The file also includes email templates that were sent to the researchers and editors we attempted to interview. It outlines the questions asked of the informants, as well as any follow-up questions. [file 12916_2023_2882_MOESM3_ESM.docx]

**Text S2. Interview protocol and questions for researchers and editors**

Two of the co-authors (AC & TJC) conducted eight semi-structured qualitative interviews with four editors and four researchers via secure video call or email between 19 July and 18 August 2022. All of the interviews were conducted in English; for seven out of eight of the participants, English was their primary working language but not their first language (i.e., native tongue). Only one participant was a native English speaker.

We first identified editors whom the research team knew personally, and who might be willing to participate in an interview. Three of these were invited to interview but did not respond or were unavailable. We then divided the list of the 302 identified journals equally and, based on which journals provided editorial email addresses on their websites, we contacted 21 for interviews. We sent emails directly to either the editor-in-chief, the managing editor, and/or the main section editor with information about our research project and a request for an interview (25 individuals were contacted at the 21 journals). We sent emails on a rolling basis until we had conducted four interviews; i.e., two male and two female editors between ages 45 and 66, who were living and working in either Europe or USA.

With the researchers, we aimed for maximum variation regarding geographical area, level of academic position, country of origin, place of work, etc. We primarily recruited interview participants via a modified version of ‘snowball sampling’^1^; rather than asking existing study participants to recruit additional participants among their acquaintances, we asked our friends and colleagues to recruit their friends and colleagues. We emailed six researchers and conducted interviews with four; one provided e-mail comments rather than participate in a video call. The researchers were comprised of three women and one man, aged between 43 and 48, all living and working in Europe (one of the female researchers is originally from and conducts some research in South America).

The interview questions were developed by (i) surveying co-authors for suggestions, and (ii) informally soliciting suggestions from other colleagues during departmental seminars. AC and TJC collaboratively decided on the text for the email invitations as well as the final interview questions and their wording. The interview guide for both editors and researchers included an informed-consent statement to ensure anonymity and to protect the identity of all interview participants. Each of the participants gave their consent to the interview, either written via e-mail and/or verbally during the video call. These calls were recorded with permission. All participants were given the right to opt out of the project at any time if they decided not to participate.

When the interviews were complete, TJC and TVV transcribed the most relevant quotes and removed any identifying features from the transcripts. AC and TJC then analyzed the empirical material in line with qualitative methodology for thematic analysis^2 3^. Here, we focused on identifying patterns across the interviews in relation to the research questions and the aims of the study. Specifically, we looked for significant points of agreement or difference between the editors and the researchers but we remained open to other notable themes that might emerge. AC, TJC, and TVV selected specific quotes for inclusion in the manuscript, and we did not include any names (either real or pseudonyms), socio-demographic information, or other identifying information in the article.

A complete overview of participants, the interview transcripts, the audio recordings, and all other project-related documents were securely stored and backed-up on the University of Copenhagen’s servers. A shared project folder was created for the project’s approved researchers to collect information about the project. The project complies with all aspects of the General Data Protection Regulation (GDPR) that is currently in effect throughout the European Union^4^. Because our research output does not contain any personal identifying information, it was not necessary to register this project with the Danish Data Protection Agency (*Datatilsynet*), the state authority that oversees the Personal Data Act^5^. However, the qualitative part of the project is in compliance with ethical guidelines for empirically-based research as outlined in the *Danish Code of Conduct for Research Integrity^6^*. The project also complies with current Danish legislation and both the University of Copenhagen’s and international guidelines for good scientific practice^7^.

**Email request for interview – EDITOR**

Dear (Journal Editor),

We are conducting a research project about manuscript-submission guidelines amongst prominent biomedical journals, and we are interested in learning about the practices at (Journal Name).

Some authors have found that the current manuscript-submission systems, processes, and guidelines are complex and difficult to navigate, which may negatively impact their experience of the submission process. It may also have financial implications and affect other aspects of their work. Thus, we are reviewing the initial submission guidelines of 300 leading biomedical journals and estimating the average time and related costs needed to comply with the diverse formatting requirements.

In order to contextualise the findings of this study, we would like to speak with the editors of select biomedical journals to gain insights into the factors that determine their specific submission guidelines. Would you be interested in participating? The interview should take no more than 15-20 minutes, and could be conducted via Zoom or Teams. If you prefer, we could also send you the questions to be answered via email.

We would like to arrange the interview/receive your answers before (DATE), so we hope to hear from you as soon as possible. Many thanks in advance for your consideration of our request.

Sincerely,

Tim Cadman and Amy Clotworthy

Tim Cadman; tim.cadman@sund.ku.dk

Amy Clotworthy; amy@sund.ku.dk

**Informed consent and interview questions – EDITOR**

*Before each interview, we will briefly introduce the purpose of the interview, the project’s preliminary research questions, and ask for their informed consent to participate. The following introduction and the subsequent interview will be audio-recorded and later transcribed.*

**Introduction**

Thank you for taking the time to participate in this interview. The interview will take about 15-20 minutes and will be semi-structured – that means that I have some specific questions to ask, but I might also inquire about other things that come up during our conversation. You can always decline to answer any question – and you are very welcome to ask about the reason for a question or anything else you may want to know.

I want to stress that this conversation is confidential, and that everyone we interview will be anonymised in terms of their name and any information that could identify a particular person. The interview will be recorded so I can transcribe the conversation later, but I will not save the files afterwards.

This main purpose of this interview is for research, but we may also use anonymous quotes in our teaching materials, conference presentations, external communication, and the like. If you later decide that you do not want to be part of this research project, you can contact me and we will delete your interview.

Do you have any questions at this point? May I have your consent to start the interview? (*Answer yes / no.*)

**Background questions**

I’m really interested in hearing your thoughts about publication guidelines and your experience working as a journal editor. But first, I would just like you to tell me a little bit about yourself.

| Socio-demographic background | |
| --- | --- |
| 1. With what gender do you identify? | - Male - Female - Non-binary - Prefer not to say |
| 1. What is your age? | ___ years |
| 1. In which country to do you live and work? |  |
| Journal-specific questions | |
| 1. How long have you had your position as a journal editor? | ___ months / years |
| 1. Is being a journal editor your full-time (paid) job? | - If no, what is your full-time job? |

**Questions for editors:**

1. Could you describe the process at your journal when you receive an article for review?

2. How well do you think the current submission process works at your journal?

3. What are your thoughts on having a universal guideline on how to prepare a manuscript across all journals?

4. How would your work tasks be impacted if submission guidelines were standardised / uniform across all journals?

*Follow-up topics –*

5. Possibility of zero guidelines on first submission/style guidelines after acceptance

6. Who makes decisions about changes to the submission guidelines at your journal?

7. Can you suggest anyone else that we should contact for an interview?

8. May we contact you again if we have further questions?

**Email request for interview – RESEARCHER**

Dear (Researcher),

My colleagues and I are conducting a research project about manuscript-submission guidelines amongst prominent biomedical journals. Some researchers have found that the current manuscript-submission systems, processes, and guidelines can be complex and difficult to navigate, which may negatively impact their experience of the submission process. It may also have financial implications and affect other aspects of their work. So, we are currently reviewing the initial submission guidelines of 300 leading biomedical journals and estimating the average time and related costs needed to comply with the diverse formatting requirements.

In order to contextualise the findings of this study, we would like to speak with researchers at different levels to gain insights into how these submission guidelines may impact them. Would you be interested in participating? The interview should take no more than 15-20 minutes, and could be conducted via Zoom or Teams. It would be either me or my colleague (Amy/Tim) who would conduct the interview. If you prefer, we could also send you the questions to be answered via email.

We would like to arrange the interview/receive your answers before (DATE), so we hope to hear from you as soon as possible. Many thanks in advance for considering our request.

Best regards – Amy / Tim

Tim Cadman; tim.cadman@sund.ku.dk

Amy Clotworthy; amy@sund.ku.dk

**Informed consent and interview questions – RESEARCHER**

Thank you for taking the time to participate in this interview. You can always decline to answer any question – and you are very welcome to ask about the reason for a question or anything else you may want to know about our research project.

I want to stress that this interview is confidential, and that everyone we interview will be anonymised in terms of their name and any information that could identify a particular person. If you answer these questions in a written document, these files will not be saved afterwards.

This main purpose of this interview is for research, but we may also use anonymous quotes in our teaching materials, conference presentations, external communication, and the like. If you later decide that you do not want to be part of this research project, you can contact me and we will delete your interview.

May I have your consent to participate in the interview? (*Please answer yes / no.*)

**Background questions**

I’m really interested in hearing your thoughts about publication guidelines and your experience with submitting manuscripts. But first, I would just like you to tell me a little bit about yourself.

| Socio-demographic background | |
| --- | --- |
| 1. What is your job title? | - Professor - Associate professor - Assistant professor / postdoc - PhD scholar - Master’s / Bachelor’s student - Retired - Other _________ (please specify) |
| 1. What is your primary discipline / research specialisation? | - _________ (please specify) |
| 1. What is your age? | - ___ years |
| 1. With what gender do you identify? | - Male - Female - Non-binary - Prefer not to say |
| 1. In which country to do you live and work? | - _________ (please specify) |

**Questions for researchers**

1. Which biomedical journals do you typically submit to (*specific titles*)? What criteria do you use to decide which journals to submit to?
2. Could you describe what happens when you submit an article to a journal – with regards to following the journal’s style guidelines, formatting, etc.?
3. What happens after an article gets rejected – i.e., what process do you go through to re-format a manuscript according to another journal’s style after a rejection?
4. If you could change one thing with current overall/general submission (guidelines) practices, what would it be?
5. How many times do you typically have to reformat and resubmit an article after a rejection (*estimate*)?
6. How much time do you use in reformatting and resubmitting an article each time it is rejected (*estimate*)?
7. What are your thoughts on having a universal guideline on how to prepare a manuscript across all biomedical journals? Or perhaps a “format-free” first submission (with specific formatting required only after acceptance)?
8. May we contact you again if we have further questions?

**References**

1. Naderifar M, Goli H, Ghaljaie F. Snowball sampling: A purposeful method of sampling in qualitative research. *Strides in development of medical education* 2017;14(3)

2. Madden R. Being ethnographic: A guide to the theory and practice of ethnography: Sage 2017.

3. Braun V, Clarke V. What can “thematic analysis” offer health and wellbeing researchers?: Taylor & Francis, 2014:26152.

4. European Parliament. GDPR is in effect: now you decide on your digital privacy, <https://www.europarl.europa.eu/news/en/headlines/society/20180522STO04023/gdpr-is-in-effect-now-you-decide-on-your-digital-privacy> (2018, accessed 8 June 2021).

5. Justice DMo. Act on supplementary provisions to the regulation on the protection of natural persons with regard to the processing of personal data and on the free movement of such data (the data protection act. Act No. 502, 2018.

6. Danish Ministry of Higher Education and Science (Uddannelses- og Forskningsministeriet). Danish code of conduct for research integrity, <https://ufm.dk/en/publications/2014/the-danish-code-of-conduct-for-research-integrity> (2014, accessed 8 June 2021).

7. University of Copenhagen Practice Committee (Praksisudvalget). Rules and guidelines, <https://praksisudvalget.ku.dk/english/rules_guide/> (2021, accessed 8 June 2021).
